# Electrocardiogram Derived QRS Duration >120 ms is Associated With Elevated Plasma Homocysteine Levels in a Rural Australian Cross-Sectional Population

**DOI:** 10.1097/MD.0000000000001080

**Published:** 2015-07-13

**Authors:** Yvonne Lee Yin Leng, Yuling Zhou, Honghong Ke, Herbert Jelinek, Joel McCabe, Hassan Assareh, Craig S. McLachlan

**Affiliations:** From the Rural Clinical School, Faculty of Medicine, University of New South Wales (UNSW), Sydney, NSW, Australia (YLYL, YZ, JM, HA, CSM); Department of Cardiology, The First Affiliated Hospital of Guangxi Medical University, Nanning, China (HK); and School of Community Health, Centre for Research in Complex Systems, Charles Sturt University, Albury, NSW, Australia (HJ).

## Abstract

Homocysteine levels in the low to moderate range for cardiovascular risk have been previously associated with left ventricular cardiac hypertrophy (LVH). Electrocardiogram (ECG) derived QRS duration has also been used as an epidemiological screening marker for cardiac hypertrophy risk. QRS duration cut offs have not been previously modeled to assess homocysteine levels in community populations. Our aims are to determine if QRS duration is associated with an elevated homocysteine level in a cross-sectional Australian aging rural population.

A retrospective study design utilizing a rural health diabetic screening clinic database containing observational data from the period January 9, 2002 till September 25, 2012. One hundred seventy-eight individuals (>21 years of age) from the database were included in the study. Inclusion criteria included being nondiabetic and having both a QRS duration measure and a matching homocysteine level within the same subject. All participants were from the Albury-Wodonga area, with a mean age of >64 years for both sexes.

Mean population homocysteine plasma levels were 10.4 μmol/L (SD = 3.6). The mean QRS duration was 101.8 ms (SD = 17.4). Groups were stratified on the basis of QRS duration (≤120 ms [n = 157] and >120 ms [n = 21]). QRS duration subgroup (≤120 ms vs >120 ms) mean differences across homocysteine levels were 10.1 μmol/L (SD = 3.3) and 12.2 μmol/L (SD = 4.7), respectively (*P* = 0.016). Other ECG parameters (PQ interval, QTc interval, and QT dispersion) measurements were not significantly associated with differences in plasma homocysteine (*P* = not significant).

We conclude that in community populations homocysteine may be moderately elevated when QRS durations are >120 ms. Small additional increases in homocysteine levels may suggest a risk factor for ECG diagnosis of LVH.

## INTRODUCTION

Epidemiological studies exploring cardiovascular risk have been modeled on the basis of QRS duration >100, >110, and >120 ms.^1–5^ In the Framingham Heart Study, electrocardiogram (ECG) derived QRS duration of >120 ms has been shown to be associated with left ventricular cardiac hypertrophy (LVH).^4^ Similarly QRS duration >120 ms is associated with increased left ventricular (LV) mass.^1,6^ Myocardial hypertrophy can lead to both prolonged LV tissue repolarization and slowing of conduction velocity.^7,8^ Slowing of conduction velocity through the cardiac ventricles is marked by QRS interval widening.^8^ Conduction slowing in the setting of cardiac hypertrophy may include impaired coronary vasodilator reserve, decreased capillary density, altered gap junction expression, increased individual cardiac myocyte cell diameter.^9^ Interestingly these pathological changes in LVH and associated conduction slowing have been demonstrated in mice models of hyperhomocysteinemia.^10^

In community studies with moderate levels of increased homocysteine, it has been shown that there is a female gender dependent association with LV mass.^11,12^ There have been no studies we are aware of to suggest whether an increased QRS duration is associated with an increased homocysteine level. Our aims are to determine if a QRS duration >120 ms is associated with an elevated homocysteine level in an Australian rural aging population.

## METHODS

### Study Population and Design

Retrospective cross-sectional analysis was performed on 178 participants’ subjects ages 21 years and over. Data were derived from a community health screening clinic database at the Albury-Wodonga campus of Charles Sturt University from January 9, 2002 to September 25, 2012. Participants in the study were from Albury-Wodonga and surrounding districts on the New South Wales-Victoria border (Southeast Australia). Inclusion criteria included being nondiabetic and having both a QRS duration measure and a matching homocysteine level within the same subject. Participants with missing homocysteine or QRS duration values were excluded from the study analysis. Written informed consent was obtained from each recruited subject. The research protocol was approved by institutional review board at Charles Sturt University.

### Data Collection

Demographic information was collected for age, smoking history, sex, height, and weight (to calculate body mass index [BMI]) and the estimated amount of exercise in hours per week. Clinical history documenting pre-existing cardiovascular disease, being treated for hypertension (or being hypertensive during study screening—see below) and Type 2 diabetes status were recorded. Study participants were also classified as having diabetes if HbA1c measurement were ≥6.5%.^13^ We retrospectively extracted data from the community health screening program and performed a cross-sectional.

### Blood Pressure

Using a standard mercury sphygmomanometer (Welch Allyn Australia (Pty) Ltd) blood pressure measurements were performed in a seated position. The blood pressure cuff placed on the upper arm, and the arm supported at the height of the heart, following a resting period of 5 min. Blood pressure measurements repeated twice (1 min apart) in each subject and the average was recorded.

### 12-Lead ECG

Resting 12-lead ECGs were obtained from each subject using a Welch Allyn PC-Based ECG system. This permitted automated QRS duration calculation from a 10 s epoch.

### Biochemistry

Venous blood was collected into ethylenediaminetetraacetic acid (EDTA)-tubes to avoid coagulation, serum separating tubes (SST) and sodium citrate (SC) tubes to measure coagulation. The EDTA-treated samples were immediately separated into plasma and red cells by centrifugation for 15 min at 800*g* and 4 °C. T Fasting blood glucose levels were measured using an Accu-Chek Advantage II glucometer (Roche Australia P/L). HbA1C, total cholesterol, triglycerides, high-density lipoprotein (HDL) and low-density lipoprotein (LDL) were measured at South West Pathology Albury. Total homocysteine (tHcy) in plasma measured via the Abbott Homocysteine (HCY) assay, via an automated fluorescence polarization immunoassay (FPIA) (Abbott Diagnostics).^15^

### Statistical Analysis

Descriptive data for continuous variables are reported as means and standard deviations (SD). Student *t* test used to compare subgroups with continuous variables and a chi-squared test for analysis of discrete variables. *P* value < 0.05 is considered statistically significant. Logistic regression analyses were used to estimate the association of covariates in relation to QRS duration. All analyses were conducted using STATA 13 (StataCorp. *Stata Statistical Software: Release 13*. College Station, TX: StataCorp LP; 2013).

## RESULTS

The baseline characteristics for 178 adult subjects are described in Table 1. Subgroup populations were stratified on the basis of QRS duration and are reported in Table 1. Fifty-five percent of study subjects were women with a mean age of 64.3 years and 42.6% were men with a mean age of 66.4 years; 34.2% of the study cohort met the criteria for type 2 diabetes and more than half of the study cohort were (59.7%) hypertensive.

**TABLE 1:**
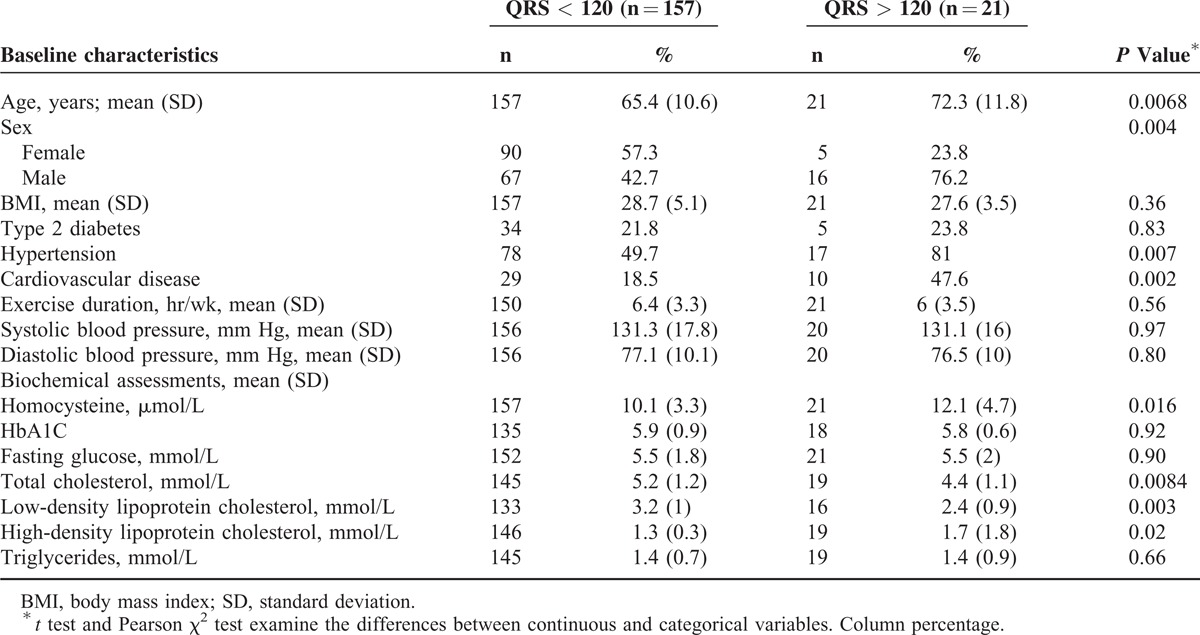
Demographics and Biochemical Characteristics of Patients by QRS Duration (<120 and >120 ms)

**FIGURE 1:**
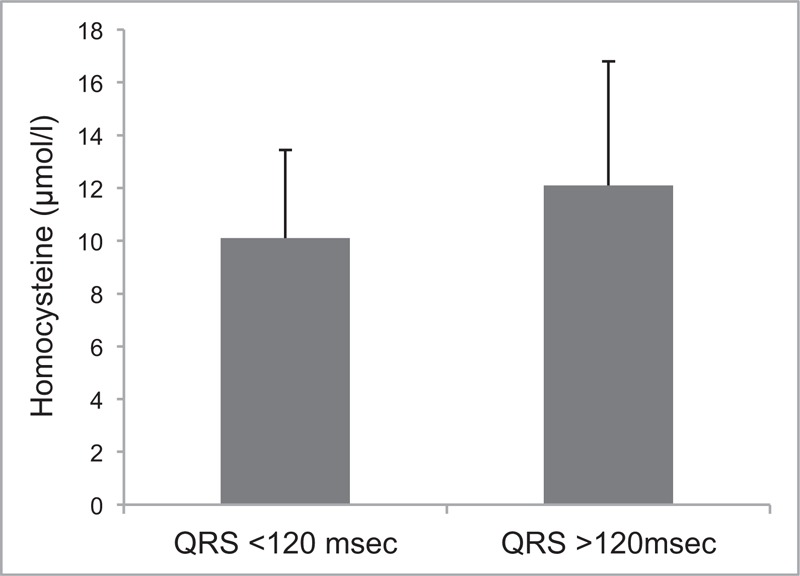
Mean differences between stratified QRS duration (≤120 ms [n = 157] and >120 ms [n = 21]; *P* = 0.016).

We report a mean population homocysteine plasma level of 10.4 μmol/L (SD = 3.6) for our 178 participants. The mean QRS duration for our 178 participants was 101.8 ms (SD = 17.4). Stratification of QRS duration into 2 sub-groups; ≤120 ms (n = 157) and >120 ms (n = 21), resulted in mean differences in homocysteine levels of 10.1 μmol/L (SD = 3.3) and 12.2 μmol/L (SD = 4.7), respectively (*P* = 0.016) (Figure 1). On the other hand, mean plasma homocysteine levels were not significantly different across subgroups when QRS duration cut-offs were re-adjusted, for example a QRS ≤100 ms vs QRS >100 ms (*P* = 0.26) or QRS ≤110 ms vs QRS >110 ms (*P* = 0.076).

Significant differences were noted in the population frequencies across the natural homocysteine quartiles. Chi-squared analysis showed that the last QRS duration quartile (≥108 ms) had an increased proportion of subjects with increased homocysteine levels (*P* = 0.032). Other ECG parameters (PQ interval, QTc interval, and QT dispersion) modeled on increasing homocysteine quartiles resulted in nonsignificant findings, apart for QRS left axis deviation (*P* = 0.035).

We performed both univariate and multivariate logistic regression to quantify the effect of covariates (demographics and biochemical measures) on QRS duration. In the univariate analysis, we found for every increase in 1 μmol/L in homocysteine, there was a 15% increase risk of a QRS duration >120 ms (*P* = 0.016). QRS duration was significantly associated with aging, male gender, a history of hypertension, and cardiovascular disease, while no significant association was found for exercise duration, BMI, and individual systolic or diastolic blood pressure measures (Table 2). After adjustment for these significant confounders the association between homocysteine and QRS duration was no longer present.

**TABLE 2:**
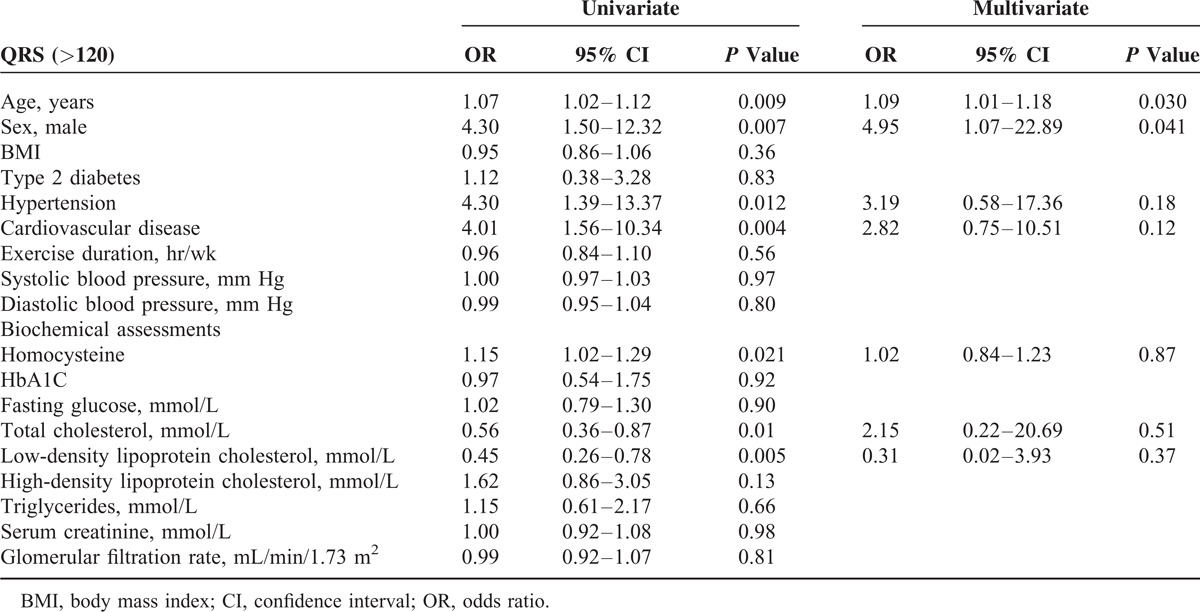
Univariate and Multivariate Logistic Regression to Examined Effects of Covariates on QRS > 120 ms

## DISCUSSION

Epidemiological evidence from community studies suggests that a physiological homocysteine plasma level above 9 μmol/L is associated with cardiovascular risk.^14,15^ Moderate cardiovascular risk is associated with plasma homocysteine levels in a range from 9.1 to 19.9 μmol/L, and high cardiovascular risk >20 μmol/L.^14^ Our mean population homocysteine levels (10.4 μmol/L) are slightly higher than a New South Wales (NSW) Central Coast aged cohort (9.39 μmol/L), where a significant proportion of the participants were hypertensive.^16^ Logistic models in the NSW Central Coast study did not reveal a significant interaction with blood pressure and homocysteine levels.^16^ We found that a previous history of hypertension influenced QRS duration (the primary outcome measure) in our studies, although when modeled as an interactive factor in regression models (with age and gender), the association with homocysteine and QRS duration was reduced. On the other hand, our subgroup consisted of only 21 participants with a QRS duration >120 ms, therefore we cautiously report our adjustments with respect to demographic clinical history in regression models and do not overstate their significance.^17–19^ We also noted in our models that neither systolic nor diastolic blood pressure measurement adjustments affected QRS duration and thus were not modeled to explore interactions with homocysteine.

When our population were stratified into 2 groups on the basis of QRS duration >120 ms or ≤120 ms, the QRS >120 ms subgroup demonstrated an increase in mean plasma homocysteine levels (12.2 μmol/L vs 10.1 μmol/L). Previous subgroup analysis conducted in Framingham Offspring Study has demonstrated an association between LVH and homocysteine levels.^12^ In this community-based sample, mild elevations in plasma homocysteine were directly related to LV mass and wall thickness in women but not in men.^12^ In our study, males were 3 times more likely to have a QRS >120 ms than females and we did not find a significant difference between mean homocysteine levels across females and males (10.2 μmol/L vs 12.9 μmol/L). The Framingham Offspring Study confirmed cardiac hypertrophy on cardiac echocardiography, while we have suggested the presence of cardiac hypertrophy on the basis of QRS duration alone, ie, >120 ms. An additional Framingham substudy has demonstrated that QRS >120 ms is a useful marker to screen for LVH in community populations.^4^ QRS duration cut offs for identifying cardiac hypertrophy have been the subject of investigation for many decades. Fragola and associates^20^ demonstrated that in LVH mean QRS duration is slightly greater >100 ms, while other investigators have also used a cut off >100 ms to predict cardiovascular events in community populations.^21^ When we explored mean homocysteine levels across subpopulations using a QRS cut off duration of between 100 and 120 ms these mean homocysteine levels were no different to participants with QRS durations <100 ms.

Numerous biochemical pathways exist in which homocysteine may influence cardiac conduction via hypertrophic signaling and or electrical remodelling.^22–24^ For example, experimental models of hyperhomocysteinemia are associated with myocardial pathology including increased ventricular fibrosis and eventual cardiac hypertrophy. Biochemical reductions in available copper and increased transforming growth factor beta 1 (TGF-β1) signaling may contribute to cardiac conduction, cardiac fibrosis, and cardiac remodelling.^25^ Additionally homocysteine in cardiac bypass and arthritis has been shown to be associated with the inflammatory cytokine IL-18 (interleukin 18),^26^ IL-18 can independently promote cardiac hypertrophy via increasing cardiac cell dimensions.^27^ In mice, chronic hyperhomocysteinemia induces ventricular dilatation via induced matrix metalloproteinase-2 and matrix metalloproteinase-9 myocardial expression changes and decreased expression of connexin 40, 43, and 45.^10^ The net results for chronic hyperhomocysteinemia in mice are atrioventricular conduction delays and a prolongation of QRS duration.^10^ In mice studies, both QTc and QTd have been shown to be affected,^10^ whereas in our study we found no evidence for an increase in either QTc or QTd across the natural quartiles of homocysteine in our population. In human transplanted hearts, there is also no significant correlation with homocysteine levels and QTc, QTcD, and QTc circadian dynamics.^28^

The reason for the increased homocysteine levels in the present study were not determined and likely to be multifactorial, including but not limited to genetics, diet, vitamin status, and renal function.^15,22^ We did not have sufficient estimated glomerular filtration rate (eGFR) measures available to explore interactions between QRS duration and homocysteine. eGFR while a leading modifiable factor for homocysteine our mean levels were only in a mild range. We also note that Dervisoglu and associates^29^ have explored eGFR and homocysteine and LVH interactions and reported no statistical significant associations. Additionally, the primary outcome measure for stratification across subgroups was QRS duration in our study and not homocysteine. Determinants for a prolonged QRS duration >120 ms are as expected including hypertension, age, and male gender in logistic models. In community-based studies, males are more likely to represent an independent factor that predicts LVH.^30,31^ Our results in a rural environment validate the Framingham Offspring Study suggesting moderate homocysteine levels are marginally elevated in cases of suspected LVH using an ECG QRS duration >120 ms as a cut-off. Unlike the Framingham Offspring Study, our study was skewed with a higher frequency of male subjects with elevations in homocysteine.
